# Identification of Genes Contributing to the Virulence of *Francisella tularensis* SCHU S4 in a Mouse Intradermal Infection Model

**DOI:** 10.1371/journal.pone.0005463

**Published:** 2009-05-08

**Authors:** Konstantin Kadzhaev, Carl Zingmark, Igor Golovliov, Mark Bolanowski, Hua Shen, Wayne Conlan, Anders Sjöstedt

**Affiliations:** 1 Department of Clinical Microbiology, Clinical Bacteriology, and Laboratory for Molecular Infection Medicine Sweden (MIMS), Umeå University, Umeå, Sweden; 2 DynPort Vaccine Company LLC, CSC, Frederick, Maryland, United States of America; 3 National Research Council Canada, Institute for Biological Sciences, Ottawa, Canada; Columbia University, United States of America

## Abstract

**Background:**

*Francisella tularensis* is a highly virulent human pathogen. The most virulent strains belong to subspecies *tularensis* and these strains cause a sometimes fatal disease. Despite an intense recent research effort, there is very limited information available that explains the unique features of subspecies *tularensis* strains that distinguish them from other *F. tularensis* strains and that explain their high virulence. Here we report the use of targeted mutagenesis to investigate the roles of various genes or pathways for the virulence of strain SCHU S4, the type strain of subspecies *tularensis*.

**Methodology/Principal Findings:**

The virulence of SCHU S4 mutants was assessed by following the outcome of infection after intradermal administration of graded doses of bacteria. By this route, the LD_50_ of the SCHU S4 strain is one CFU. The virulence of 20 in-frame deletion mutants and 37 transposon mutants was assessed. A majority of the mutants did not show increased prolonged time to death, among them notably Δ*pyrB* and Δ*recA*. Of the remaining, mutations in six unique targets, *tolC*, *rep*, *FTT0609*, *FTT1149c*, *ahpC*, and *hfq* resulted in significantly prolonged time to death and mutations in nine targets, *rplA*, *wbtI*, *iglB*, *iglD*, *purL*, *purF*, *ggt*, *kdtA*, and *glpX*, led to marked attenuation with an LD_50_ of >10^3^ CFU. In fact, the latter seven mutants showed very marked attenuation with an LD_50_ of ≥10^7^ CFU.

**Conclusions/Significance:**

The results demonstrate that the characterization of targeted mutants yielded important information about essential virulence determinants that will help to identify the so far little understood extreme virulence of *F. tularensis* subspecies *tularensis*.

## Introduction

*Francisella tularensis* is a facultative intracellular bacterium that is highly virulent and highly contagious and one of the agents given highest priority in the offensive biological weapons programs of the US and Soviet Union during the Cold War [1]. Besides this notoriety, it is most often encountered in nature as the etiological agent of a potentially lethal disease, tularemia [2]. It occurs in a wide variety of mammals, most frequently in small rodents, hares, and rabbits. In humans, it is a rare disease in most countries but epidemic outbreaks occur in certain areas of the world, *e.g.*, in Finland, Russia, and Sweden [3]. Often it is spread via vectors such as ticks, biting flies or mosquitoes. Besides vector bites, it is capable of infecting the hosts via a variety of other routes such as inhalation, direct contact, ingestion of contaminated food or water. The most serious form of tularemia is contracted via inhalation [4]. Regardless of how the infection was established, the target organs are draining lymph nodes, liver, spleen, and lung. The pathology in these organs appears to be similar in rodents and humans and therefore the mouse model has become a commonly used experimental model [5].

In 1959, it was recognized that *F. tularensis* isolates show distinct virulence differences for many host species [6]. In particular, some strains unique to North America demonstrated very high virulence for most animal species tested and they gave rise to very serious forms of clinical disease with very high mortality [7]. This subspecies is designated *F. tularensis* subspecies *tularensis*, or type A. In humans and experimental animals, doses of 10 bacteria or less can cause severe infection and the mortality of human respiratory tularemia caused by such strains may be 30% or higher without proper antibiotic treatment [8]. Epidemiological information gathered recently indicated that there are two variants of subspecies *tularensis*, AI and AII, and that the former is responsible for the most serious types of tularemia [9]. The less virulent subspecies of *F. tularensis* encountered in Europe, Asia, as well as North America is named *holarctica*, also type B. The subspecies is often associated with semi-aquatic rodents [7]. In Europe, tularemia is most frequently observed in hares and rodents.

Due to re-emergent concerns about its potential use as a weapon of mass destruction by terrorists, the development of *Francisella* vaccines has been given high priority [10]. During the 1940's and 50's, extensive efforts were undertaken in the Soviet Union to develop a live vaccine and several attenuated vaccine strains were generated by repeated passages of a subspecies *holarctica* strain on media containing serum which led to an irreversible attenuation. One of these was transferred to the USA, and was used to derive the current live vaccine strain (LVS) [10]. In humans, LVS administered by scarification confers excellent protection against systemic challenge with high doses of subspecies *tularensis* and against lower doses of inhaled bacteria [10]. Therefore, development of an improved vaccine against aerosol exposure has become an important goal. It has been postulated that protection against the highly virulent type A subspecies might be optimal if the vaccine is based on a homologous strain containing potentially protective antigens missing from subspecies *holarctica* [10]. Until a few years ago, a targeted mutagenesis strategy for *F. tularensis* was hampered by limitations in the available genetic tools and selection markers. However, several examples of targeted mutations in the prototypic SCHU S4 strain have recently been reported. The targets include the O-antigen locus *wbtDEF*, *purMCD*, *katG*, *FTT0918*, *iglC*, *FTT1103*, and *dsbB* [11], [12], [13], [14], [15], [16]. All of these mutants, with the exception of *katG*, showed variable degrees of attenuation, but most of them *e.g.*, *wbtDEF*, *iglC*, *purMCD*, and *dsbB* appeared to confer little or no protection against a challenge with wild-type SCHU S4 [11], [12], [14], [15]. Immunization with *FTT0918* afforded solid protection against intradermal challenge and a partial protection against a low-dose aerosol challenge with the parental strain [14]. However, Δ*FTT0918* showed an LD_50_ of 10^4^–10^5^ CFU which is less than LVS which has an LD_50_ of >10^6^ CFU. The only example so far of a markedly attenuated strain that confers protection appears to be Δ*FTT1103* [16]. Thus, there is a shortage of attenuating targets that still preserve the immunogenicity of the corresponding deletion mutants. One drawback in the work to rationally identify such targets has been our rudimentary knowledge regarding the virulence mechanisms of *F. tularensis*. It is phylogenetically distant from all other human pathogens, and consequently a high proportion of its genes are unannotated and predictions of its virulence pathways in *silico* are uncertain [17]. Moreover, many mechanisms, such as type III and type IV secretion systems, essential for the virulence of many other intracellular bacteria are missing in *F. tularensis*. Much research has been focused on the role of a large pathogenicity island of some 20 genes that is present in two copies in all *F. tularensis* genomes except that of *F. novicida* [18]. Among these, in particular, the intracellular growth locus (*igl*) operon has been studied intensively since it appears to be essential for the intracellular replication of the bacterium [19], [20], [21], [22], [23], [24], [25], [26]. Also, the *pil* system has been the focus of much research [27], [28], [29], [30] and also the *F. tularensis* mechanisms for acquisition of iron, leading to the identification of the *fsl* operon [31], [32]. Despite the recently rapid progress of *F. tularensis* research, much remains to be understood about the pathogenicity of this enigmatic bacterium. One way to address this will be to characterize the phenotypes of targeted mutants since it will yield important information regarding pathways essential and non-essential for the virulence of *F. tularensis*. We have investigated the contribution to virulence of various *F. tularensis* genes or pathways by creating transposon or deletion mutants and testing their virulence in the well-established mouse model. Here we describe the findings related to the generation of 20 deletion and 37 transposon mutants.

## Results and Discussion

Throughout the manuscript, the term virulence factor will be used in accordance with the definition proposed by Wood et al; “A component of a pathogen that when deleted specifically impairs virulence but not viability” [33]. We observed impaired growth of the *purL* and *purF* mutants in Chamberlain's broth but not on agar plates (Table 1). The *kdtA* mutant showed delayed multiplication at 6 h but reached a high density after 24 h whereas all other mutants described herein showed a growth pattern indistinguishable from SCHU S4 regardless of medium used (Table 1). Therefore, we find the use of the term virulence factor to be appropriate when the corresponding mutants showed an attenuated phenotype, with the exception of the three aforementioned mutants.

**Table 1:** 

Mutant strain	Bacterial growth^a^	
	6 h	24 h
SCHU S4	1.75^b^	≥2.00
Tn-*purF* c	0.29	0.29
Tn-*purL*	0.29	0.30
Tn-*kdtA*	1.74	≥2.00
Tn-*rplA*	1.45	1.90
Δ*iglB*	1.70	≥2.00
Δ*iglD*	1.75	≥2.00
Δ*ggt*	1.78	≥2.00
Δ*kdtA*	0.86	1.62
Δ*wbtI*	1.57	≥2.00
Δ*glpX*	1.62	1.88
control	0	0

a An overnight culture of the indicated strain was inoculated in Chamberlain's medium to an OD_600_ of 0.2. Data shown is from one representative experiment out of three.

b The optical density (OD_600_) at the indicated time point is shown.

c Tn indicates that the strains are transposon mutants.

Using a standard procedure for transposon insertion mutagenesis of SCHU S4 [34], a total of 743 mutants were generated by screening on plates containing kanamycin. Of these, 159 were characterized by sequencing of the targeted gene. Some of the insertion mutants were analyzed for virulence in the mouse model based on intradermal (ID) administration of graded doses of bacteria. In this model, even the lowest inocula of <10 CFU of SCHU S4 invariably resulted in a lethal infection [35]. Since one aim of the work was to identify attenuating targets that can serve as the basis for generation of live vaccine candidates, the goal was to avoid mutations that conferred marginal attenuation and therefore the first screen was performed based on the administration of a dose of 1,000 CFU. In total, 37 transposon mutant strains were screened. Only strains with insertions in annotated genes with defined biological roles were included and these are listed in Table 2. The screen identified a number of attenuating targets, most markedly the following genes; *FTT0141* (*rplA*), *FTT1455c* (*wbtI*), *FTT1720c* (*purL*), *FTT1721c* (*purF*), and *FTT1561* (*kdtA*), which all had an LD_50_≥10^3^ CFU.

**Table 2:** Transposon mutant strains included in this study.

Designation	Gene target	Degree of attenuation (LD_50_)	Insertion location^a^	Function	References
*rplA*	*FTT0141*	>10^3^ CFU	19	50S ribosomal protein L1	
*purF*	*FTT1721*	>10^7^ CFU	17	Amidophosphoribosyl-transferase	[42], [44]
*purL*	*FTT1720c*	>10^7^ CFU	10	phosphoribosylformylglycinamide synthase	[40], [42], [44]
*kdtA*	*FTT1561*	>10^3^ CFU	1	3-deoxy-D-manno-octulosonic-acid transferase	
*wbtI*	*FTT1455*	>10^3^ CFU		LPS O-antigen synthesis	
*tolC*	*FTT1724c*	<10^3^ CFU*	39	outer membrane efflux protein	
*FTT0609*	*FTT0609*	<10^3^ CFU*	69	Unannotated	
*rep*	*FTT1087c*	<10^3^ CFU*	22	UvrD/REP superfamily I DNA and RNA helicases	[41]
*lon*	*FTT0626*	<10^3^ CFU	76	DNA-binding, ATP-dependent protease La	[41], [43]
*tig*	*FTT0623*	<10^3^ CFU	69	trigger factor (TF) protein (peptidyl-prolyl cis/trans isomerase), chaperone	[43]
*ppdK*	*FTT0250*	<10^3^ CFU	45	phosphoenolpyruvate synthase/pyruvate phosphate dikinase	
*FTT0069*	*FTT0069c*	<10^3^ CFU	12	Unannotated	
*FTT1640c*	*FTT1640c*	<10^3^ CFU	39	activator of osmoprotectant transporter ProP, fragment	[44]
*tet*	*FTT0444*	<10^3^ CFU	73	drug:H+ antiporter-1 (DHA1) family protein	[43]
*pilO*	*FTT1158c*	<10^3^ CFU	46	Type IV pili glycosylation protein	
*htpX*	*FTT0862c*	<10^3^ CFU	50	Zn-dependent protease with chaperone function	
*asd*	*FTT0425c*	<10^3^ CFU	30	aspartate semialdehyde dehydrogenase	
*prfC*	*FTT0118*	<10^3^ CFU	37	peptide chain release factor 3	[44]
*fslE*	*FTT0026c*	<10^3^ CFU	28	siderophore uptake	
*trpB*	*FTT1773c*	<10^3^ CFU	27	tryptophan synthase beta chain	
*hslU*	*FTT0687c*	<10^3^ CFU	63	ATP-dependent protease HslVU, ATPase subunit	
*yjjK*	*FTT1782c*	<10^3^ CFU	80	ABC transporter ATP-binding protein	[43], [44]
*usp*	*FTT0245*	<10^3^ CFU	20	universal stress protein	
*omp26*	*FTT1542c*	<10^3^ CFU	36	protein of unknown function	
*elbB*	*FTT0654*	<10^3^ CFU	90	DJ-1/PfpI family protein	[41], [43]
*ipdC*	*FTT1744c*	<10^3^ CFU	8	indolepyruvate decarboxylase	
*FTT0156*	*FTT0156*	<10^3^ CFU	25	Unannotated	
*rnhB*	*FTT1278c*	<10^3^ CFU	81	ribonuclease HII	
*glpA*	*FTT0132*	<10^3^ CFU	27	glycerol-3-phosphate dehydrogenase	
*uvrA*	*FTT1312c*	<10^3^ CFU	68	DNA excision repair enzyme, subunit A	[43]
*trpE*	*FTT1802c*	<10^3^ CFU	35	anthranilate synthase component I	[41]
*tgt*	*FTT1120c*	<10^3^ CFU	4	queuine tRNA-ribosyltransferase	
*FTT0891*	*FTT0891*	<10^3^ CFU	73	Conserved hypothetical membrane protein	[44]
*cphA*	*FTT1130c*	<10^3^ CFU	65	Cyanophycin synthetase	[44]
*aroC*	*FTT0876c*	<10^3^ CFU	1	Chorismate synthase	[41]
*mutL*	*FTT0486*	<10^3^ CFU	96	DNA mismatch repair enzyme with ATPase activity	
*moxR*	*FTT0290*	<10^3^ CFU	37	MoxR-like ATPase	
*bipA*	*FTT1179*	<10^3^ CFU	66	GTP-binding translational elongation, factor Tu and G family protein	[41], [43]

* extended time to death, >7 days.

a in % of gene length from 5′ end.

In *F. tularensis*, *rplA* (FTT0141), encoding the 50S ribosomal protein L1, and *rplK*, encoding the 50S protein L11, are predicted to be transcribed by the same operon. Also in *Corynebacterium glutamicum*, the *rplK* and *rplA* genes have been identified as co-transcribed and required for the activation of the RelA protein [36], the source of the initiator of the stringent response, (p)ppGpp, an adaptation to amino acid starvation [37]. Even if *F. tularensis* harbors both *relA* and *spoT* homologues, two enzymes which appear to substitute for each other in many cases, it is possible that expression of the *rpl* operon still is required in order to activate the stringent response and, thus, the attenuation of the *rplA* mutant may depend on an impaired adaptation to amino acid starvation. The relevance of the findings regarding the *kdtA*, *wbtI*, and *pur* mutants are discussed later.

**Figure 1:**
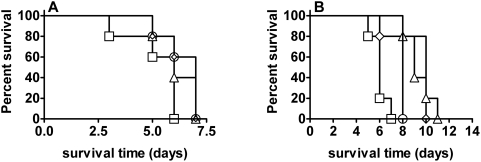
Survival of transposon and deletion mutants of SCHU S4. In A) BALB/c mice were challenged intradermally with 1000 CFU of SCHU S4 (square), Δ*FTT0609* (triangle), Δ*tolC* (circle), or Δ*rep* (diamond). In B) BALB/c mice (n = 5/group) were challenged intradermally with 10 CFU of SCHU S4 (square), Δ*FTT1149* (diamond), Δ*ahpC* (triangle), or Δ*hfq* (circle).

Among the tested Tn mutants, a challenge of 10^3^ CFU with three additional strains, *FTT1724c* (*tolC*), *FTT0609*, *FTT1087c* (*rep*), resulted in significantly longer mean survival of 7 days compared to 5 days for mice infected with SCHU S4 (Figure 1A). The function of TolC of *F. tularensis* LVS has been investigated and it was proposed to be involved in a multidrug resistance machinery, however, it showed no defective replication in murine bone marrow-derived macrophages but significant attenuation of virulence in a mouse model [38]. It was hypothesized that it functions as part of a type I secretion system. Our present findings indicate that in SCHU S4, TolC makes a marginal contribution to the virulence.

*FTT0609* encodes a peptidase of the M24 family protein. The homologues have not been described as virulence determinants but a similar peptidase has been identified in *Lactobacillus delbrueckii* and found to be a dipeptidase active on X-proline dipeptides [39]. *FTT1087c* (*rep*) encodes an ATP-dependent DNA helicase that never has been described as a virulence factor.

It should be remarked that the interpretation of the results with these mutants can be confounded by some inherent ambiguities of the transposon insertion strategy. First, the insertions may lead to polar effects if the targeted gene is located in the upstream part of an operon. Thereby, the phenotype observed may depend on the disrupted or impaired transcription of another gene of the operon. Second, a lack of phenotype cannot unambiguously rule out an important role of the target since the location of the insertion may be downstream of a biologically active site. The exact locations of the insertions are indicated in Table 2. Third, they might exhibit more overt attenuation at lower challenge doses.

Notably, two out of the five mutants identified in our analysis as markedly attenuated, *rplA* and *kdtA*, were not identified in any of five studies intended as whole-genome screens of virulence factors of *F. tularensis* [40], [41], [42], [43], [44]. Three of these studies were based on *F. novicida* [40], [41], [44] and one on *F. tularensis* LVS [43] and some of the differences may relate to the use of other model organisms than SCHU S4. Also, administration routes may affect the results, since two of the studies employed inhalation models [41], [43]. The only previous study using SCHU S4 assessed the intracellular replication in a hepatic cell line but not growth *in vivo* [42]. The targets included in the present study that had been identified in any of the five previous studies as virulence factors are listed Tables 2 and 3. Of the 30 transposon mutants that did not show any degree of attenuation or extended time to death, 13 had been identified in one or more of the aforementioned four studies as virulence factors. The most likely reason that we did not identify them is that the degree of attenuation in SCHU S4, if any, is less than the 10^3^ CFU used for challenge. Also, since eight were identified in only the studies based on inhalation models, the route of administration and the use of other model organisms may be contributing factors.

**Table 3:** Deletion mutant strains included in this study.

Designation	Gene target	Degree of attenuation (LD_50_)	Deleted region^a^ [total number of codons]	Function	References
*ΔiglB*	*ΔFTT1358*, *ΔFTT1713*	>10^7^ CFU	61–354 [515]	intracellular growth locus	[43], [44]
*ΔiglD*	*ΔFTT1356*, *ΔFTT1711*	>10^7^ CFU	1–125+RBS [402]	intracellular growth locus	[41], [44]
*ΔRD5*	*ΔFTT0522-0523*	<10^1^ CFU	30(*FTT0522*)–380 (*FTT0523*) [326–409]	type I restriction-modification system	
*ΔRD8*	*ΔFTT1071*	<10^1^ CFU	29–140 [169]	anti restriction-modification system	
*ΔoppD*	*ΔFTT0125*	<10^1^ CFU	13–299 [323]	peptide/opine/nickel uptake transporter, PepT family proteins	
*ΔchiA*	*ΔFTT0715*	<10^1^ CFU	3–756 [761]	chitinase, glycosyl hydrolase family 18	
*ΔFTT1149*	*ΔFTT1149*	<10^1^ CFU*	16–482 [503]	amino acid-polyamine-organocation (APC) superfamily	
*ΔpyrB*	*ΔFTT1665*	<10^1^ CFU	2–297 [307]	aspartate carbamoyltransferase	[42], [44]
*ΔrecA*	*ΔFTT1750*	<10^1^ CFU	14–351 [360]	recombinase A protein	[41], [43]
*ΔmdaB*	*ΔFTT0961*	<10^1^ CFU	23–175 [197]	recombinase A protein	[41]
*Δgpx*	*ΔFTT0733*	<10^1^ CFU	13–143 [157]	glutathione peroxidase	
*ΔahpC*	*ΔFTT0557*	<10^1^ CFU*	3–160 [175]	peroxiredoxin of the AhpC/TSA family	
*ΔsodC*	*ΔFTT0879*	<10^1^ CFU	23–178 [186]	superoxide dismutase (Cu-Zn) precursor	
*ΔrimK*	*ΔFTT0178*	<10^1^ CFU	28–489 [490]	glutathione synthase/ribosomal protein S6 modification enzyme	
*Δggt*	*ΔFTT1181*	>10^5^ CFU	4–591 [602]	gamma-glutamyltranspeptidase	[42]
*ΔkdtA*	*ΔFTT1561*	>10^7^ CFU	53–351 [432]	3-deoxy-D-manno-octulosonic-acid transferase	
*ΔwbtI*	*ΔFTT1455*	>10^7^ CFU	40–319 [361]	sugar transamine/perosamine synthetase	
*Δhfq*	*ΔFTT0630*	<10^1^ CFU*	3–96 [110]	host factor I for bacteriophage Q beta replication	
*ΔpckA*	*ΔFTT0449*	<10^1^ CFU	11–527 [531]	phosphoenolpyruvate carboxykinase	
*ΔglpX*	*ΔFTT1631*	>10^7^ CFU	19–312 [329]	fructose 1,6-bisphosphatase II	[41], [43], [44]

* extended time to death, >7 days.

a localization of the deleted region with regard to the codon number of the encoding gene.

All 20 mutants described henceforth were generated as in-frame deletions of the target genes. All investigated mutants are listed in Table 3. *F. tularensis* strains harbor a 31.2 kb locus, designated the *Francisella* pathogenicity island (FPI), containing some 20 genes, many of which appear to execute functions necessary for its intracellular life cycle [18]. The bacterium initially resides in a phagosome before it escapes into the cytoplasm via degradation of the phagosomal membrane. This is followed by extensive replication in the cytosol leading to apoptotic death of the macrophage and bacterial egress [45]. Among the FPI, the genes encoded by the *igl* locus have attracted most attention. It has been suggested that *iglA* and *iglB* genes contribute to a type VI secretion system although direct proof of a functional secretion system is lacking [21]. Functions encoded by the operon appear to be essential for the phagosomal escape and intracellular growth, since disruptions or deletions of FPI encoded genes of the low virulent strains *F. tularensis* LVS and *F. novicida*, such as *iglA*, *iglB* [21], [46], *iglC* [22], [23], [25], [26], and *iglD* [19], [47], all are predominantly contained within the phagosome and unable to replicate intracellularly. Therefore, the findings collectively suggest that their functions are critical for the early stages of *F. tularensis* intracellular trafficking. These defects lead to highly attenuated phenotypes *in vivo* since the *iglC* mutants of LVS and SCHU S4 are essentially avirulent in mice [14], [22]. Moreover, we previously observed that immunization with **Δ** *iglC* conferred only a marginal degree of protection against a subsequent challenge with a strain of subspecies *tularensis* and hypothesized that this is due to its limited replication *in vivo* [14].

In agreement with our previously published results on **Δ** *iglC* [14], we here observed that both the **Δ** *iglB* and **Δ** *iglD* mutants showed marked attenuation (Table 3) but provided only marginal protection against SCHU S4 challenge (data not shown). Similar to our recent findings based on the same mutants in LVS [19], [46], the results suggest that expression of each of the IglB, IglC, and IglD proteins is essential to the virulence of *F. tularensis* and the absence of any one of them leads to very significant attenuation even of the highly virulent strain SCHU S4.

Since information on 15 *F. tularensis* genomes is present in public databases, there is ample amount of genetic information available for comparative genomic studies. This has resulted in the identification of a number of regions exclusively present in genomes of subspecies *tularensis* strains. The first description of such regions identified eight so called regions of difference (RD), altogether comprising 21 open reading frames [48]. One of these regions, RD5, contained two ORFs, FTT0522 - FTT0523, that display similarity to a type I restriction-modification system present in *Shewanella oneidensis* and a type I restriction enzyme from *Bacteroides thetaiotamicron*. A second region, RD8, contained an 11.2 kb region unique to subspecies *tularensis* strains, however, at least two of the eight ORFs were predicted to be pseudogenes. *FTT1071* is among the intact RD8 genes and it shows similarity to a gene encoding an antirestriction protein from *Nitrosomonas europaea*. Moreover, the region designated RD2 contains *oppD* (FTT0125) which is a unique gene in subspecies *tularensis* strains, since a 960 bp internal deletion in subspecies *holarctica* strains has resulted in a fused ORF with a disrupted *oppD* coding sequence [48]. Mutants lacking the gene/genes of each of the three regions were constructed but each of the mutants showed full virulence in the mouse model, killing mice at an intradermal dose of 10 CFU with no extended time to death compared to SCHU S4 (Table 3). Therefore, our results did not in any way indicate that these type A-unique genes contribute to the high virulence of the subspecies.

An extensive comparative genomic analysis by Rohmer et al identified 35 genes that displayed unique sequence variations changing the protein content of the corresponding proteins in LVS compared to other investigated *F. tularensis* strains [49]. Of these 35 genes, 15 were predicted to have lost the function of the encoded proteins and seven of the latter were found to be under strong selective pressure. Among the seven, *FTT1149*, an amino acid transporter family protein, and *FTL1521* (*chiA*), a chitinase family 18 protein were found. The latter protein was found to be over-expressed by subspecies *tularensis* during *in vivo* growth, and secreted *in vitro* by *F. novicida* by a type IV pilus secretion system [28]. Secreted chitinases are essential for the establishment of the arthropod infection by malaria parasites [50] and therefore their presence in *F. tularensis* may be of biological relevance. However, we could not detect any attenuation of the *chiA* mutant (Table 3). Thus, this chitinase appears to play no role in the mouse model by the intradermal route but since *F. tularensis* is an arthropod-borne bacterium, it is possible that it plays a role in tularemia spread via such vectors. The *FTT1149* mutant demonstrated no increased LD_50_ in as much as a dose of 10 CFU uniformly killed mice, but the mean time to death was significantly prolonged (Figure 1B). Thus, the predicted loss of function of this gene in LVS may make a marginal contribution to its attenuation.

Mutations of *pur* genes often confer marked attenuations as exemplified by *purL* mutants in *Brucella abortus* [51] and *purE* in *B. melitensis* [52]. As aforementioned, when screening a library of transposon mutants in the mouse model, we identified attenuated mutants with an insertion in the *purL* as well as the *purF* genes. Both genes function in the early stage of purine biosynthesis. Both of these mutants were found to be considerably attenuated as mice survived inocula of 10^7^ CFU (Table 2). Mice that survived after administration of 10^5^ CFU were challenged intradermally with 10 CFU of SCHU S4 but they did not show any extended time to death compared to naïve mice.

The same two mutants, as well as additional *pur* mutants, were identified in a screen performed by Qin et al when they searched for transposon mutants that showed defective intracellular replication [42]. Thus, an intact purine metabolic pathway appears to be very important for the growth intracellularly and *in vivo* of *F. tularensis*. This is not surprising since it appears to be the case for many other pathogens [51], [52], [53], [54]. Extensive work has been carried out in *F. tularensis* characterizing a *purMCD* mutant of SCHU S4 as well as of LVS [12]. Both showed marked attenuation and protected against intradermal challenge with the parental strain, but there was no protection against intranasal challenge [12]. The effects of *pur* mutations have been assessed also in subspecies *novicida* and it was found that immunization with a *purF* mutant provided protection against a challenge with wild-type *novicida* whereas *purA*, *purCD*, *purL*, and *purM* mutants were not attenuated and did not protect mice against a homologous challenge [40], [55]. Neither a *purF* nor *purA F. novicida* mutant afforded protection against a challenge with SCHU S4 [55]. Thus, similar to our results, the findings indicate that the attenuation conferred by defects in the many of the purine metabolism genes does not yield mutants that are capable of inducing protective immunity. One reason may be that marked attenuation leads to shorter overall survival of the mutants and thereby limited induction of protective T cells. The findings may be analogous to those observed when infection is terminated by antibiotic treatment. North et al reported that abridging a *Listeria* infection with antibiotic treatment resulted in a quick loss of protective T cells from the spleen and they concluded that the onset and duration of decay of the protective response was determined by the number of replicating bacteria in the tissues [56]. Thus, mutations, such as those in *igl*, *wbt* and *pur* genes that significantly impair the bacterial survival *in vivo* may lead to much less vigorous priming of protective T cells.

Mutations in pyrimidine biosynthesis pathways have been investigated in many bacterial species and generally they confer attenuation. In fact, also in *F. tularensis* SCHU S4, the pyrimidine pathway has been demonstrated to be important for intracellular growth, since a *pyrB* transposon mutant of SCHU S4 showed decreased growth rate in HepG2 cells [42]. It was also reported that infected mice showed an extended time to death after administration of low doses of the mutant although they all died after administration of 200 CFU [42]. We observed no survivors with an inoculum of 10 CFU of a *pyrB* deletion mutant indicating that the attenuation was marginal at most (Table 3). This indicates that *in vivo* the genes needed for the generation of pyrimidines, at least *pyrB*, are not as essential as those needed for purine synthesis.

Central to the survival of pathogenic microorganisms is the ability to withstand the stress conferred by reactive oxygen metabolites since these are important defence mechanisms expressed by infected mammals, in particular by the oxidative burst of phagocytic cells. These defense mechanisms include enzymes that detoxify reactive oxygen species, such as superoxide dismutase (Sod), catalase (KatG), alkyl hydroperoxide reductase (AhpC), glutathione peroxidase (GpX) and DNA repair systems (Rec), which repair damage resulting from oxidative stress. Additional protective mechanisms involve proteins that reduce the quinone pool using NADPH as the reductant, *e.g.*, MdaB. Previously, we observed that a KatG mutant of LVS and SCHU S4 showed significantly increased susceptibility to hydrogen peroxide [13]. However, only the LVS mutant demonstrated significant attenuation. In the present study we found that SCHU S4 *gpX*, *sodC*, *mdaB*, and *ahpC* mutants demonstrated no increased LD_50_ although *ahpC* showed a significant prolonged time to death at a challenge dose of 10 CFU (Figure 1B). Collectively, our results demonstrate that *F. tularensis* possesses a variety of means to compensate for loss of major oxidative stress-combating factors and that the important enzymes have overlapping functions. The compensatory mechanisms are sufficient to allow replication so that the specific mutants exhibit at most marginal attenuation *in vivo*. However, considering the extended time to death, it is likely that the *ahpC* mutant, like the previously described *katG* mutant [13], has an impaired rate of replication in target organs indicating an important function for AhpC. Notably, despite five conjugations, no mutants with combined deletions of *ahpC* and *katG* were isolated. This may indicate that the combined mutations conferred a compromised phenotype with low viability.

To assess the relative importance of DNA repair systems, *recA* was deleted. The *recA* mutant did not demonstrate any significant attenuation in the mouse model. The *F. tularensis* RecA has been recombinantly expressed and shown to suppress the sensitivity of the carrier to DNA-damaging agents in *E. coli*, thereby confirming an important role of the enzyme [57]. Thus, it appears likely that *F. tularensis* possesses additional DNA repair systems to compensate for the absence of RecA. The findings are similar to those on *recA* mutant of *Mycobacterium bovis* BCG [58]. It was found that the mutant had intact virulence for mice and a normal dormancy response, *i.e.*, the entry into a non-replicative persistent phase as an adaptation to a decrease in oxygen tension.

Hfq is a bacterial RNA chaperone involved in the posttranscriptional regulation of many stress-inducible genes via small noncoding RNAs and found to be essential for expression of virulence in several bacteria, *e.g.*, uropathogenic *E. coli* and *Pseudomonas aeruginosa* [59], [60]. It also appears to play important roles for resistance to reactive nitrogen and oxygen radicals [59]. In *F. tularensis*, a recent study found that an Hfq mutant of a subspecies *holarctica* strain contributed to its stress tolerance and was attenuated in a mouse model [61]. In the latter case, the difference was found using a competition infection experiment whereas the difference in average time to death was at most 2 days. We observed that mice infected with 10 CFU of Δ*hfq* lived significantly longer than those infected with SCHU S4 although the former mice all died within 8 days (Figure 1B). Thus, the role of Hfq appears to be at most marginal for the virulence of SCHU S4 but considering that the difference compared to the parental strain was marginal also in subspecies *holarctica*, the contribution of Hfq may not be distinct between subspecies *tularensis* and *holarctica*.

Gluconeogenesis in the absence of pyruvate requires the functions of phosphoenolpyruvate carboxykinase (PEPCK), encoded by *pckA*, which catalyses the decarboxylation and phosphorylation of oxaloacetate to form phosphoenolpyruvate (PEP) [62]. The enzyme can also perform the conversion of PEP to oxaloacetate to maintain the TCA cycle. PEPCK is thought to be of particular importance when organisms are exploiting fatty acids as their major carbon source and it was found to be important in mycobacteria since a *pckA* mutant of BCG was attenuated both with regard to intramacrophage growth and in mice [63]. On the other hand, it is dispensable in *Salmonella enterica* [64]. In the latter species, even a *ppsApckA* double mutant showed only a marginal degree of attenuation indicating that gluconeogenesis plays, at most, a minor role for *Salmonella* virulence. The gene *ppsA* encodes for phosphoenolpyruvate synthase, which produces PEP from pyruvate. In *Salmonella*, the combined deletions completely block gluconeogenesis above pyruvate [64]. We observed no attenuation of the Δ*pckA* mutant (Table 3) but whether its role is in the gluconeogenic pathway for carbohydrate formation, or in the conversion of PEP to oxaloacetate to maintain the TCA cycle, remains to be determined. However, we observed that a *glpX* mutant demonstrated marked attenuation. Although the exact function of the *F. tularensis* GlpX has to be determined experimentally, in *E. coli*, the enzyme converts fructose-1,6-bisphosphate to fructose-6-phosphate (Fbp) as part of the gluconeogenesis but *E. coli* also possesses a second enzyme, fructose-1,6-bisphosphatase (*fbp*), that carries out the same reaction and, in fact, GlpX has been shown to be functional only when overexpressed [64]. Since there seems to be no Fbp homologue in *F. tularensis*, it is possible that GlpX is the sole enzyme responsible for the conversion to fructose-6-phosphate in the species. The extreme attenuation of the SCHU S4 mutant of GlpX, which showed an LD_50_ of >10^7^ CFU (Table 3), suggests that the gluconeogenesis is critical for the full virulence of *F. tularensis in vivo*.

Our screening of transposon mutants identified several additional attenuated strains. Based on these findings, deletion mutants were generated and their virulence assessed. One of these targets was γ-glutamyl aminotranspeptidase (Ggt). It has a major role in glutathione metabolism and is responsible for the first step in the degradation pathway of glutathione, and transfers a γ-glutamyl moiety from glutathione to amino acids or uses it as a source for cysteine. It can also contribute to the synthesis of glutathione through the transfer of γ-glutamyl peptides. A transposon mutant was also identified by Qin et al as attenuated for intracellular growth in a hepatocyte cell line and in macrophages [42] and recently by Alkhuder et al. which showed that *F. tularensis* LVS, in fact, utilized glutathione and gamma-glutamyl-cysteine dipeptide as cysteine sources to ensure intracellular growth [65]. We observed that the SCHU S4 mutant had an LD_50_ of ≥10^5^ CFU (Table 3). Thus, it is likely that also *F. tularensis* SCHU S4 utilizes glutathione during its intracellular growth. Since glutathione is abundant in the cytosol, it is a rational mechanism to ensure an intracellular supply of γ-glutamyl moieties and cysteine. Also in *Helicobacter pylori*, a crucial role of Ggt has been identified since a mutant was attenuated in both piglets and mice [66].

The RimK protein of *Escherichia coli* has been isolated and characterized as a protein that adds glutamic acid residues to the carboxyl terminus of ribosomal protein S6 of the organism [67]. The protein family including *E. coli* RimK encompasses a number of proteins with ATP-dependent carboxylate-amine ligase activity, where acylphosphate intermediates are involved in the catalytic mechanism [68]. Moreover, it was demonstrated that a *rimK* mutant in the extreme thermophile *Thermus thermophilus* demonstrated a lysine auxotrophic phenotype [69] but this could represent an adaptation to extreme living conditions, and may have no relevance for human pathogens. Since it could be predicted that RimK may contribute to ribosomal functions and many times ribosomal dysfunctions lead to attenuation, we included it in the study but no attenuation was observed (Table 3).

The LPS of *F. tularensis* constitutes a unique molecule characterized by minimal endotoxicity [70]. The *F. tularensis* core oligosaccharide chain from LPS is not well characterized but the bacterium possesses a *kdtA* gene which in other bacteria is known to encode a 3-deoxy-D-manno-2-octulosonic acid (Kdo) transferase [71]. The enzyme adds Kdo residues to the lipid A portion of the LPS. A *kdtA* mutant of *Moraxella catharralis* demonstrated enhanced clearance in lungs and nasopharynx in a mouse aerosol model [72]. Similarly, we observed that the SCHU S4 Δ*kdtA* deletion mutant showed marked attenuation (Table 3), but the immunized mice did not show any extended time to death when they 5 to 6 weeks later were challenged by ID or aerosol administration of approximately 10 CFU of SCHU S4. The same results were obtained with a SCHU S4 Δ*wbtI* mutant, a gene of the lipopolysaccharide O-antigen synthesis. Three previous studies have studied the properties of *wbt* mutants with regard to intracellular replication and virulence in a mouse model [11], [73], [74]. In agreement with our results, *wbtI* mutant of SCHU S4, *wbtDEF* mutants of SCHU S4, LVS and *F. novicida* were attenuated after intradermal challenge of mice. However, none of them afforded protection against a homologous challenge [11]. Thus, it appears as if lipopolysaccharide mutations in general and irrespective of *F. tularensis* subspecies give rise to highly attenuated bacteria with minimal immunogenicity.

The strategy used herein, to screen targeted mutants for marked attenuation in a mouse model has several caveats that have to be considered when interpreting the results. One is the extreme virulence of strains of subspecies *tularensis* in the mouse model. All evidence indicates that one CFU is sufficient to cause a lethal disease [35]. Thus, the level of attenuation has to be marked before a significant increase in the LD_50_ can be observed. This is evidenced by our previous study of the KatG mutant of SCHU S4 that was found to be as virulent as the parent strain with regard to the LD_50_ although it showed markedly decreased bacterial numbers in liver and spleen [13]. Normally, for other *F. tularensis* strains, a very good correlation is found between the peak number of CFU and outcome of infection [75]. The high virulence of SCHU S4 most likely means that peak numbers of CFU have to be several log_10_ lower before attenuation is evidenced as an increased LD_50_. Another concern is a lack of complementation. We have analyzed LVS mutants with in-frame deletions in each of the four *igl* genes and our recently published data showed that there were no significant effects of the expression of the three other proteins in each of the four mutants and each of the phenotypes could be complemented [19], [46]. Thus, the in-frame deletions did not confer polar effects on downstream genes and, therefore, the effects of such deletions appear to be specific to the target genes, supporting the notion that our findings in the present study directly reveal if the target genes have an essential function.

Most studies on *F. tularensis* are carried out using the model organisms *F. novicida* or LVS. As we demonstrated previously for KatG, and in the present study for ChiA and TolC, the role of many proteins are distinct in the model organisms compared to the highly virulent subspecies *holarctica* or *tularensis* strains. Therefore, the data from studies on *F. novicida* or LVS cannot simply be extrapolated and assumed to be relevant for highly virulent strains.

There are some general caveats regarding the use of an *in vivo* screening model as a tool to search for virulence factors. If a specific mutation has no effect on virulence, the finding does not necessarily mean that the disrupted target plays no role in virulence since an alternative pathway can compensate for the defect. Moreover, virulence gene expression may depend on specific signaling, and, thus, attenuation might be the effect of repression of virulence factors. Another caveat is that a specific defect might play an important role at one stage of infection but not at another, which is not revealed by the screening method, *e.g.*, the inactivated target can play a role by a route of inoculation not employed. Since analysis of virulence factors in whole organisms remains complex because of the aforementioned caveats, our results have to be interpreted with some caution, but nonetheless, the presented data will be important for our understanding of virulence mechanisms utilized by this highly virulent intracellular bacterium.

## Materials and Methods

### Bacterial strains

The *F. tularensis* strain SCHU S4 (subspecies *tularensis*) was obtained from the *Francisella* Strain Collection (FSC) of the Swedish Defence Research Agency, Umeå. The descriptions of the mutants of SCHU S4 that were used in the study are listed in Tables 2 and 3.

All *in vitro* bacteriological work was carried out in a biosafety level 3 facility certified by Swedish Work Environment Authority and also certified to be Select Agent compliant by the Centers for Disease Control and Prevention, USA. Bacteria were routinely cultivated on GC II Agar Base supplemented with IsoVitalex at 37°C in 5% CO_2_ overnight.

### Construction of mutant strains

The upstream and downstream regions flanking each target gene were amplified by means of Phusion DNA polymerase (Finnzyme) and primer pairs (Table 4) were designated so that one primer for the upstream region and one primer for the downstream region contained 5′end sequences complementary to each other to provide “sticky” sequences for the following overlapping PCR. The flanking regions were 1,000–1,500 bp for all constructs except the ones for *rimK*, *chiA*, and *FTT1149* that were between 500 and 600 bp. The final PCR product was cloned to pGEM-Teasy vector (Promega), and introduced in *E. coli* TOP10 strain (Invitrogen), then subsequently re-cloned to the pDMK2 vector using *NotI* sites and propagated in the strain *E. coli* S17-1λ*pir*. pDMK2 (GenBank accession no. FJ824848) is a derivative of pDMK [13] with additional restriction sites in the former. The resulting pDMK2 plasmid containing the deletion construct was conjugatively transferred from S17-1 to SCHU S4 as described previously [76]. Clones with the respective derivative of pDMK2 integrated into the SCHU S4 chromosome by a single homologous recombination event were selected on plates containing kanamycin and polymyxin B, and the legitimate integration was verified by PCR. Clones with correct integrations were then subjected to sucrose selection. This procedure selected for a second cross-over event in which the integrated plasmid, encoding *sacB*, was excised from the chromosome. Kanamycin-sensitive clones were examined by PCR confirming the deletion of the genes and the exact location verified by sequencing. The exact sizes of the deletions are indicated in Table 3. All deletions were in-frame, thereby not affecting transcription downstream.

**Table 4:** 

Gene	Primer sequence 5′ – 3′			
	OF^a^	IR	IF	OR
Δ*iglB*	ccggtcgacggggtt taggattttaaaaca	aagtgataactccataaggtttctagcattgta	gaaaccttatggagttatcacttgcaaatatt	aagagttaatagcgggaagtcat
*ΔiglD*	ggcattctagaaactaagtcac	tgcagctgcatagctatggttttaaacata	aaccatagctatgcagctgcaatatatcct	gcgtgtcgacttagccgtgccaattacca
Δ*RD5*	gctagcagttaaagaaatccgtact	gtgcttttatataatttacatttagcaaaccatcatta	ctaaatgtaaattatataaaagcactagaacaacaa	ctcgagtaaacttagccattagccaaatt
Δ*RD8*	gctagccagacttgatgatttgattcatc	ggattgacgcagactttgaatctctagctcgt	attcaaagtctgcgtcaatccatttaccgtat	ctcgagccattattttgtcctaaaccag
Δ*RD2*	agcagatggagatagtggtttt	gcttagtatcgctgatatctctaaccgcaga	gagatatcagcgatacta agccagaattgatttc	gctggcttactctatacgcc
Δ*chiA*	tcagtctagataccatccatagagaatacgcc	aactacttgtgatggtacattgttgatacttaggac	aatgtaccatcacaagtagttacataccaaggcgc	atgtctcgagagtaaatctccaaggcaatg
Δ*FTT1149*	actgctgcggctttactagcgggtaatact	ataacgcttatcggcactaaacaacgacatcttttttacaga	ttgtttagtgccgataagcgttatgtcgcattctgtagatct	cgcaaacataccacctaaaacacccaatat
Δ*pyrB*	aagaggagctagatgcttatattaagacta	agccaatagcgtcattttttactcctaaaacgttctatttat	gagtaaaaaatgacgctattggctttattagtaatatcctaa	agtgtcatactagttacactaggctttgac
Δ*recA*	ggtttctgcaactacagcta	gagtaactgcctgtgataaggctgattcta	cttatcacaggcagttactcaagatgagct	atgtccaattgcaactatct
Δ*mdaB*	cagcaccatgcaagctagtat	cgttgataagttgattcaaagcaccttttga	tttgaatcaacttatcaacgaatttacgtta	agcgtattagctgatatttgt
Δ*gpx*	gtccaccttgctataaactgcttgat	taaatcttctggccatcatttgcagttagattaaa	gcaaatgatggccagaagatttaattccagatatt	tctagattggcgcaccttctattc
Δ*ahpC*	aaggttactttgtagaagata	gagaagactcagtcatgtctaactcctttgt	agacatgactgagtcttctccagaaaactta	gtaacgttcgagttaactcta
Δ*sodC*	ctagctagccacaagtggttactgtaaa	tccacaccatgtgcagtctgctaatacaagt	gcagactgcacatggtgtggagttatagcagactaa	ccgctcgagaatcgctaagcacgttgca
Δ*rimK*	gacgatacgacttagctgccttgagtaata	agttgagctttagtgggttgtttctagatttgaataaggata	gaaacaacccactaaagctcaactaacagatcttcaagcact	gcttattttgcggagattctagctctatga
Δ*ggt*	ctttggttcaaaaggtttgtgttcatcatt	gctagctctgcgacgacgcattaaagaaacaaaaataaacca	ttaatgcgtcgtcgcagagctagcgcattagctataggttat	ccgcctattaaacttacgcttgagtatgct
Δ*kdtA*	ccagagctcgctgatgaaattggacttagt	ttgctaatgcggcgaatctctctgcccat	agagattcgccgcattagcaaagccaatt	ccggtcgactcaccctcaatatctagttgt
Δ*wbtI*	ccagagctcaattgcttcccgcattgttac	gttagtgcaagagccaaagcagtatatgcc	ctgctttggctcttgcactaacggaccat	ccggtcgacgctttattagcagataaacta
Δ*hfq*	agcttttggtaaagattttaatgattatgt	aatttcaagatctgacattgtctcacttccctttaattattt	gagacaatgtcagatcttgaaattcaagagaatgaaggtaat	acgaaacttggcttgatattttctaattta
Δ*pckA*	cgcttacttgtaattgtgtaa	aaataattggctcatctatggttgaaggatc	catagatgagccaattatttagacgctaatt	acgtaccatcactgcaattac
Δ*glpX*	gggcccaaggtagtgagcttataggt	gtgctctgctagtgctgccagctcagtaa	ctggcagcactagcagagcacagttttgactt	gtcgacagctagcgctcctgataa

a OF: Outer Forward primer, IR: Internal Reverse primer, IF: Internal Forward primer, and OR: Outer Reverse primer. Recombination arms were first generated using OF+IR and IF+OR primer pairs and subsequently ligated by PCR with OF+OR primers.

### Screening for transposon insertion mutants

The EZ-Tn5<*ori*V/KAN-2>Insertion Kit (Epicentre Biotechnologies, Madison, WI) was used to create random transposon insertion mutations in *F. tularensis* SCHU S4. The transposome reaction mixture was prepared by mixing either the EZ::TN <kan-2> transposon (Epicentre) or the kan-Ft transposon with transposase in accordance to the Epicentre protocol with the addition of an incubation at −20°C for at least 48 h. Kan-Ft is derivative of Kan-2 containing an *F. tularensis* codon-optimized version of *aphA* expressed under control of a modified *F. tularensis groEL* promoter. The plasmid containing kan-Ft was kindly provided by Dr Tom Kawula [77]. Electrocompetent *F. tularensis* cells were prepared essentially as described by Kawula et al. [34] and 1 µl of the transposome was used for electroporation into *F. tularensis* at 3.0 kV, 25 µF, and 200 ohms in an 0.1 cm electroporation cuvette. Immediately after electroporation, the cells were suspended in 0.4 ml Chamberlain medium, incubated for 4 h at 37°C, and then plated on GC II Agar Base supplemented with IsoVitalex containing 10 µg/ml of kanamycin. After overnight incubation, individual kanamycin-resistant colonies were identified. Chromosomal DNA was prepared by use of the DNAEasy Blood & Tissue Kit (Qiagen, Hilden, Germany) according to the manufacturer's instructions. Transposon insertion sites in the bacterial chromosome were determined by direct sequencing of genomic DNA using Kan-2 FP-1 (ACCTACAACAAAGCTCTCATCAACC) and Kan-2 RP-1 (GCAATGTAACATCAGAGATTTTGAG) for the Kan-2 transposon or FtKmFP (GGCATGCAAGCTTGCCAACGA) and FtKmRP (TCGAGCCAATATGCGAGAACAC) for the KanFt transposon. For sequencing, the BigDye Terminator Kit (Applied Biosystem, Foster City, CA) was used. Two to five µg of chromosomal DNA and 10 pmol primer were used and the reactions were carried at 95°C for 1 min followed by 60 cycles of 10 s at 95°C, 5 s at 55°C, and 4 min at 60°C. The transposon insertion site was determined by aligning the sequence to the *F. tularensis* SCHU strain genome database (www.Francisella.org). All mutants included in the study were unambiguously identified by this analysis.

### Infection of mice

Mouse challenge experiments were performed at the National Research Council of Canada, Institute for Biological Sciences in a federally-licensed small animal containment level 3 facility that is also approved by the NIH for Select Agent research. Specific-pathogen-free female BALB/c mice were purchased from Charles River Laboratories (St. Constant, Que.). Mice were maintained and used in accordance with the recommendations of the Canadian Council on Animal Care Guide to the Care and Use of Experimental Animals. For intradermal inoculations, stocks of the strains were diluted in sterile saline. Actual concentrations of inocula were determined by plating. Intradermal inocula (50 µl/mouse) were injected into a fold of skin in the shaved mid-belly. All deletion mutants were initially administered as an intradermal inoculum of 10^3^ CFU. If this dose was sublethal, additional mice were challenged with 10^5^ and 10^7^ CFU. If 10^3^ CFU was lethal, the mutant was also given a dose of 10 CFU. In all cases, there were five mice per group. Mice that survived the highest intradermal challenge dose were subsequently exposed to a ∼20 CFU small particle aerosol of SCHU S4 as previously described [78]. All other survivors of mutant challenges were challenged intradermally with 10^3^ CFU of SCHU S4. Whenever feasible, mice were euthanized by CO_2_ asphyxiation as soon as they displayed signs of severe infection. In our experience such mice were at most 24 h from death, and time to death of these animals was estimated on this premise. The significance of prolonged time to death of mice was determined by using the Logrank test. A *P* value<0.05 was considered significant.
